# Spatial multi-omics guides ASCT2-targeted delivery of glycolysis inhibitor for cervical cancer suppression and chemoresistance reversal

**DOI:** 10.1016/j.jare.2025.11.035

**Published:** 2025-11-16

**Authors:** Xiaojiao Li, Xiangchuan Qin, Xiejun Zhao, Yufeng Liu, Ling Wang, Kefeng Li, Jinqiu Li, Jia Ma, Liangliang Dai, Ayshamgul Hasim

**Affiliations:** a Department of Basic Medicine, Xinjiang Medical University, Xinjiang Key Laboratory of Molecular Biology of Endemic Diseases, and Key Laboratory of High Incidence Disease Research in Xinjiang (Xinjiang Medical University), Ministry of Education, Urumqi 830011 Xinjiang, PR China; b BioBank, The First Affiliated Hospital of Xi’an Jiaotong University, Xi’an 710061 Shaanxi, PR China; c Shaanxi Engineering Research Center for Biobank and Advanced Medical Research, The First Affiliated Hospital of Xi’an Jiaotong University, Xi’an 710061 Shaanxi, PR China; d School of Pharmacy, Health Science Center, Xi’an Jiaotong University, Xi’an 710061 Shaanxi, PR China; e College of Food Engineering and Nutritional Science, Shaanxi Normal University, 620 West Chang’an Avenue, Xi’an 710119 Shaanxi, PR China; f Department of Medical Oncology, The First Affiliated Hospital, Xi’an Jiaotong University, Xi’an 710061 Shaanxi, PR China; g Xi’an Key Laboratory of Stem Cell and Regenerative Medicine, Institute of Medical Research, Northwestern Polytechnical University, Xi’an 710072 Shaanxi, PR China

**Keywords:** Spatial multi-omics analysis, Glycolysis, HKⅡ, ASCT2, Cervical Cancer, Chemoresistance Reversal

## Abstract

**Introduction:**

Cervical carcinoma (CC) mortality remains high due to chemoresistance. Targeting glycolytic reprogramming is promising since CC cells depend on enhanced glycolysis for proliferation and chemoresistance. However, clinical translation faces two barriers: the lack of spatially resolved validation of glycolytic vulnerability in CC specimens, and absence of CC-specific delivery systems for lonidamine (LND), a potent glycolytic inhibitor with poor bioavailability and hepatotoxicity.

**Objectives:**

Here, we aim to establish spatially resolved validation of tumor-associated glycolytic reprogramming and identify differentially enriched receptors within malignant regions in clinical CC specimens. Leveraging these findings, we will design a tumor-targeted nanoplatform for the precise delivery of a glycolytic inhibitor to CC cells, to achieve tumor suppression and reverse chemoresistance.

**Methods:**

Spatial metabolomics and spatial transcriptomics analyses were employed to validate tumor-associated glycolytic reprogramming and identify specifically overexpressed receptors within malignant regions of clinical CC specimens.

**Results:**

Through spatial multi-omics analysis, we demonstrated upregulated glycolysis in malignant regions of CC and identified solute carrier family 1 member 5 (ASCT2), a glutamine transporter, as a superior CC-specific surface marker compared to the pan-cancer nanocarrier targets like CD44. These findings were corroborated through multi-platform validation spanning single-cell RNA-seq dataset, TCGA cohorts, paired patient specimens, matched murine samples, and multiple CC cell lines. In this context, we designed a glutamine-functionalized liposomal system that exploits ASCT2 overexpression to enable CC-selective accumulation of LND. Our findings reveal that this nanoagonist significantly impedes CC growth by disrupting ATP supply and inducing ROS-mediated cellular damage. Moreover, this nanoagonist effectively reverses cisplatin (DDP)-induced chemoresistance in CC by inhibiting MRP2-mediated DDP efflux and blocking ribose-5-phosphate-mediated DNA repair.

**Conclusion:**

By integrating spatial multi-omics with rational nanocarrier design, we designed a glutamine-functionalized liposomal system that exploits ASCT2 overexpression for tumor-selective accumulation of LND. Our findings revealed that this nanoagonist achieves significant CC suppression and chemoresistance reversal.

## Highlights

• Spatial multi-omics validate glycolysis preference in cervical cancer (CC)
• Spatial transcriptomics reveals ASCT2 specifically overexpressed in CC.
• We developed a nanoagonist that selectively targets ASCT2 and reprograms glycolysis.
• The nanoagonist inhibits CC growth by blocking ATP supply and inducing ROS toxicity.
• The nanoagonist reverses chemoresistance by blocking DDP-efflux and DNA repair.

## Graphical abstract

The glycolysis-reprogramming nanoagonist inhibits cervical cancer (CC) and reverses chemoresistance. a) A scheme for the fabrication of the nanoagonist. b) Upon specifically targeting CC by the Gln-ASCT2 recognition, tumor-selective accumulation of lonidamine (LND) was achieved. This nanoagonist significantly attenuated the growth of cervical cancer by cutting off ATP supply and inducing ROS-mediated cellular damage. More importantly, reprogramming glycolysis by LND effectively reverses cisplatin (DDP)-induced chemoresistance in CC by suppressing MRP2-induced DDP efflux and inhibiting ribose-5-phosphate-mediated DNA repair.

**Figure:**
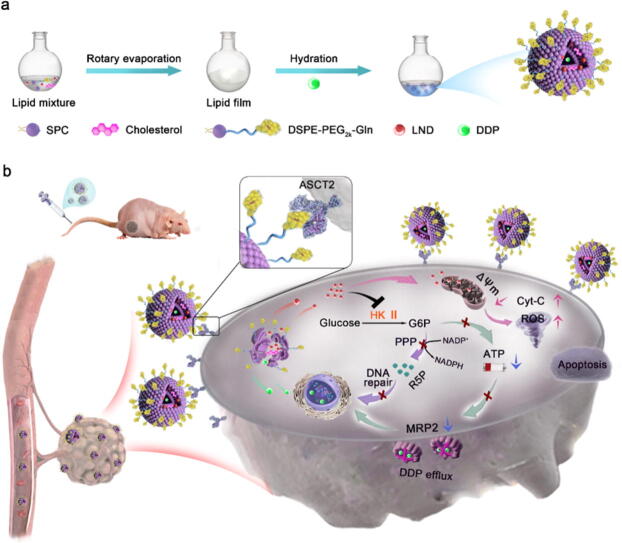

## Introduction

Cervical carcinoma (CC) remains a leading cause of female cancer mortality globally, with chemotherapy resistance posing significant therapeutic challenges [[1], [2], [3], [4]]. Targeting glycolytic reprogramming emerges as a promising strategy, as tumor cells rely on enhanced glycolysis for both proliferation and chemoresistance [[5], [6], [7], [8], [9], [10]]. Despite hyperactivated glycolysis is implicated in CC [[11], [12], [13], [14]], two critical barriers impede clinical translation. Firstly, there is a lack of spatially resolved validation of glycolytic vulnerability in CC specimens, limiting spatially targeted therapy. Secondly, while the key glycolytic inhibitor lonidamine (LND) requires CC-specific delivery due to its poor bioavailability and hepatotoxicity [14], current platforms lack CC-specific surface markers, compromising accumulation efficacy.

In this study, spatial metabolomics and spatial transcriptomics were employed to rigorously map metabolic vulnerabilities and identify an optimal CC-specific surface marker in clinical CC specimen. The spatial omics analysis confirmed a significant upregulation of glycolytic activity within tumor regions compared to adjacent non-tumor areas, solidifying glycolysis as a key therapeutic target for CC. To enable tumor-specific LND delivery in CC, we analyzed our ST dataset to identify tumor-enriched cell-surface targets. This analysis revealed that the glutamine (Gln) transporter, solute carrier family 1 member 5 (ASCT2), exhibits greater tumor-specific overexpression than conventional pan-cancer target CD44, highlighting its potential as a target to facilitate LND delivery specifically to CC tumor site. These findings were corroborated through multi-platform validation spanning single-cell RNA-seq dataset, TCGA cohorts, paired patient specimens, matched murine samples, and multiple CC cell lines.

**Fig. 1:**
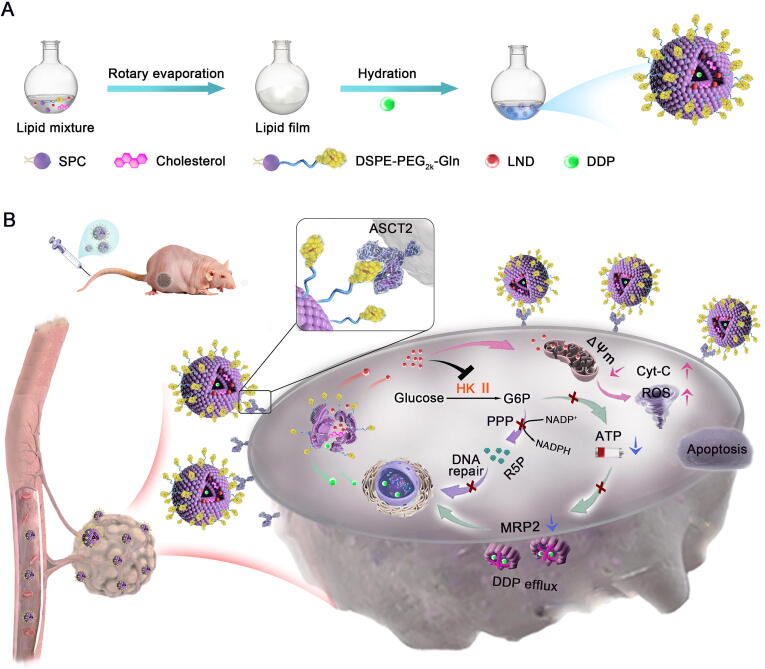
Schematic illustration of glycolysis reprogramming nanoagonist for cervical cancer inhibition and chemoresistance reversal. a) A scheme for the fabrication of the nanoagonist. b) Upon specifically targeting CC by the Gln-ASCT2 recognition, tumor-selective accumulation of lonidamine (LND) was achieved. This nanoagonist significantly attenuated the growth of cervical cancer by cutting off ATP supply and inducing ROS-mediated cellular damage. More importantly, reprogramming glycolysis by LND effectively reverses cisplatin (DDP)-induced chemoresistance in CC by suppressing MRP2-induced DDP efflux and inhibiting ribose-5-phosphate-mediated DNA repair.

Leveraging these findings, we fabricated a Gln-functionalized liposome (GL) to efficiently deliver LND to CC. Following tumor accumulation via ASCT2-mediated targeting, the released LND markedly down-regulated glycolysis, triggered the dissipation of mitochondrial transmembrane potential, and facilitated reactive oxygen species (ROS) generation, thereby cutting off the energy supply and causing cellular damage for tumor regression. Moreover, given the potential of glycolysis in chemoresistance, a cisplatin (DDP)-resistant CC model was developed to further investigate the antitumor efficacy of this nanoagnoist. Delivering LND by GL dramatically reversed chemoresistance in CC through two mechanisms: on one hand, by downregulating the expression of ATP-dependent multiple drug resistance protein 2 (MRP2), which is responsible for DDP efflux [15,16]; and on the other hand, by inhibiting the generation of ribose-5-phosphate (R5P), which is essential for DNA repair [[8], [9], [10]]. Collectively, this novel strategy offers a new perspective on CC treatment and chemoresistance reversal (Fig. 1).

## Materials and methods

### Sample collection for spatial multi-omics analysis

The study was approved by the ethics committee of the First Affiliated Hospital of Xinjiang Medical University (NO. 20220304–01). Written informed consent was obtained from the CC patient prior to the tissue collection. Sample from the patient diagnosed with cervical cancer who underwent surgery were included in this study. Freshly collected CC tissue was embedded in OCT Medium (Leica, cat. no.39475237), snap-frozen on dry ice, and stored at −80 °C. Spatial multi-omics analysis was performed by Shanghai OE Biotech Co., Ltd. (China).

### Spatial metabolomic analysis

Embedded specimens were cryosectioned at 10 μm thickness using a Leica CM1950 Microtome Cryostat (Leica Microsystems, Germany) and mounted onto charged target plates. For mass spectrometry imaging (MSI) analysis, the sections were dried at −20 °C for 1 h and then at room temperature for 2 h prior to analysis. An adjacent section was retained for hematoxylin-eosin (H&E) staining to enable spatial correlation. The MSI analysis was performed on an AFADESI-MSI platform coupled with a Q-Orbitrap mass spectrometer. The optimized ionization parameters are as follows: solvent delivery at 5 μL/min with 45 L/min nebulizing gas; spray voltage maintained at 7 kV; 3 mm dual nozzle distances. High-resolution spectra were acquired at 70 k resolution over *m*/*z* 70–1000 Da with automatic gain control targeting 2E6 ions and 200 ms maximum injection time.

Raw spectral data underwent format conversion using imzMLConverter (v3.0.19330) before processing in MsiReader (v1.02). The Cardinal package (v2.14.0)was used to perform background subtraction, followed by normalization of all mass spectrometry images to the total ion count (TIC) per pixel. Tissue-specific metabolic profiles were extracted by aligning MSI data with high-resolution H&E images. Differential metabolites were identified by screening with orthogonal partial least squares-discriminant analysis (OPLS-DA) (VIP > 1.0) and validating through two-tailed Student's t-tests (*P* < 0.05). Only metabolites meeting both criteria were retained for further analysis.

### Spatial transcriptomic analysis

Spatial transcriptomic analysis was performed using the 10x Genomics Visium platform. Briefly, 10-μm cryosections were mounted onto Visium slides for permeabilization time optimization. Selected sections were then processed on Visium Spatial Gene Expression Slides (10x Genomics, PN-1000185) for methanol fixation, HE staining, and library construction following the manufacturer’s protocols (CG000160, CG000239). Final libraries were sequenced on BGI DNBSEQ-T7 with PE100 mode.

For ST-seq analysis, FASTQ files were aligned to the GRCh38 human reference genome using Space Ranger (v1.2.0) from 10x Genomics, summarizing unique molecular identifier (UMI) counts per barcode. Tissue-overlaying spots were distinguished from background based on histological images to ensure accurate spatial mapping. Applying spatial transcriptomics quality thresholds (UMI counts > 200, genes detected > 500, mitochondrial gene percentage < 20 %), we identified 8 spots (0.16 % of total) failing criteria. Given their negligible proportion, these spots were retained in downstream analyses to maintain tissue architecture integrity. The spatial metrics showed a mean UMI count of 15,947, a mean genes detected of 3,562, and a mean mitochondrial percentage of 6 % (Fig. S1). The filtered matrix was analyzed in Seurat (v4.1.0) using SCTransform for normalization and selection of the top 3,000 highly variable genes. Principal component analysis (PCA) was conducted to reduce dimensionality. Graph-based clustering was performed to cluster cells according to their gene expression profile with the FindClusters function. Spots were visualized using a 2-dimensional Uniform Manifold Approximation and Projection (UMAP) algorithm. Differentially expressed genes (DEGs) were selected via FindMarkers (presto test; threshold: Bonferroni-adjusted *P* < 0.05, |log_2_FC|>0.58). KEGG pathway enrichment analysis of DEGs was conducted using R (version 4.0.3) with the KEGG 2021 human pathway database, employing a hypergeometric test with a significance threshold of p-value < 0.05. The top pathways were selected based on p-value ranking from the significant results. To prioritize biological relevance to cervical carcinogenesis, we excluded pathways meeting three pre-defined criteria: (1) HPV-unrelated pathogen infections (e.g., Influenza A, Tuberculosis); (2) organ-specific diseases without established links to gynecological malignancies (e.g., neurodegenerative disorders including Parkinson's/Alzheimer's); (3) broadly conserved cellular processes lacking disease-specific insights (e.g., Proteasome, Phagosome).

### Analysis of the public scRNA-seq dataset

The filtered UMI count matrix for cervical cancer scRNA-seq data (GSE208653) was obtained from GEO and analyzed using Seurat (v4.1.0). Data normalization employed LogNormalize, followed by principal component analysis (PCA) dimensionality reduction. Cells were clustered via graph-based clustering (FindClusters) and visualized using UMAP (RunUMAP). Cell type annotation utilized the celldex R package and the HumanPrimaryCellAtlasData reference, enabling epithelial cell identification and extraction. Tumor-associated and normal epithelial cells were categorized according to sample origin for comparative analysis. Differentially expressed genes (DEGs) were identified using FindMarkers (Wilcoxon rank sum test), with significance defined as Bonferroni-adjusted p-value < 0.05 and |log_2_FC| > 0.58. KEGG pathway enrichment analysis for the identified DEGs was executed in R (v4.0.3) using the hypergeometric distribution. To maintain biological relevance to cervical carcinogenesis, the same exclusion criteria used in spatial transcriptomics were applied.

### Cell lines and mice

The human cervical carcinoma cell lines SiHa and ME180 were provided by BeiNa Culture Collection (Shanghai, China). HeLa, MS751, and C33A were from the Cell Bank of Chinese Academy of Sciences (Shanghai, China). End1/E6E7 was purchased from Fuheng Cell Center (Shanghai, China). The cisplatin (DDP)-resistant SiHa cell line (designated as SiHa-DR) was developed by stepwise increasing DDP concentration and intermittent administration for 10 months. CCK-8 analysis, the expression of multidrug resistance proteins and transcriptome sequencing were employed to validate the successful establishment of SiHa-DR. All cells were maintained at 37℃ under 5 % CO_2_ and cultured in Dulbecco’s modified Eagle medium (DMEM) medium supplemented with 10 % FBS, 100 U mL^−1^ streptomycin and penicillin.

Female BALB/C nude mice (5–6 weeks) were purchased from Beijing Vitalstar Biotechnology Co, Ltd. All protocols for experiments were approved by the Ethics Committee of Xinjiang Medical University (No. IACUC–20211016–62). Mice were maintained on a 12-hour light–dark cycle at room temperature with sterile pellet food and water.

### CCK-8 assay

Cell viability was examined with the Cell Counting Kit-8 (CCK-8, Bimake). Briefly, cells were trypsinized and plated in 96-well plates at a density of 5000 cells/well. After treatment, cells were washed with PBS and incubated with 10 % CCK-8 solution for 2 h at 37℃. The absorbance at 450 nm was measured using a microplate reader (Bio-Tek, Cytation 5).

### Western blotting

Cells were collected, lysed, and analyzed by western blotting to determine the expression of the indicated proteins in each group. The following antibodies were used: rabbit anti HKⅡ (1:2500, Proteintech, YM8118), rabbit anti ASCT2 (1:1000, Immunoway YT7482), rabbit anti Bcl-2 (1:1000, Proteintech 26593–1-AP) rabbit anti Bax (1:500, ABclonal A7627), rabbit anti Cytochrome *C* (1:500, ABclonal A4912), rabbit anti P-gp (1:1000, Immunoway YT3692), rabbit anti Survivin (1:1000, ABways CY5070), rabbit anti MRP2 (1:1000, Immunoway YT2840), rabbit anti G6PD (1:1000, Abclonal A27296), rabbit anti GAPDH (1:3000, CST D16H11) and rabbit anti β-actin (1:50000, ABclonal AC026). The expression of *β-actin* or *Gapdh* gene was used as an internal control.

### Synthesis and characterization of GL

For the synthesis and characterization of Gln-PEG_2K_-DSPE, 100 mg of NHS-PEG_2K_-DSPE were dissolved in 2 mL of C_2_H_5_OH, followed by the addition of a glutamin solution (2.0 equivalents). The resulting mixture was allowed to react at 40 ℃ for 2 h and subsequently dialyzed (MW cut-off: 1000 Da) against ultrapure water for 24 h. The product was then freeze-dried under vacuum to yield Gln-PEG_2K_-DSPE in powder form. The successful synthesis of Gln-PEG_2K_-DSPE was confirmed using proton nuclear magnetic resonance (^1^H NMR) and matrix-assisted laser desorption/ionization time-of-flight mass spectrometry (MALDI-TOF-MS).

Gln-functionalized liposomes (GL) were prepared via a film dispersion method. Briefly, a mixture containing soy phosphatidylcholine (SPC), cholesterol (Chol), and Gln-PEG_2K_-DSPE at a molar ratio of 20:1:2 was dissolved in chloroform. Then, the solution was evaporated at 60 °C to remove residual chloroform, resulting in the formation of a thin lipid film. The thin lipid film was hydrated with deionized water and extruded through a mini-extruder with pore sizes of 200 nm to obtain GL. To prepare drug-loaded GL, LND was added to chloroform, and DDP was dissolved in deionized water during the synthesis of GL. Finally, the size distribution and Zeta potential of the liposomes were verified by TEM (JEOL) and Zetasizer Nano ZS (NanoBrook 90plus PALS).

### Drug loading and release properties

To assess the drug loading efficiency of GL, Cy7 was employed as a model drug. In short, GL@Cy7 was loaded into a dialysis bag and immersed in 37 ​°C PBS for continuous stirring until complete drug release. The fluorescence of Cy7 was measured using UV/vis spectroscopy at 780 nm. The loading efficiency and encapsulation efficiency were calculated according to the following formulas:

Loadingefficiency%=weightofCy7inGL/weightofGL×100%

Encapsulationefficiency%=weightofCy7inGL/weightoffeedingCy7×100%

Next, the real-time drug release behavior of GL was determined. Briefly, 300 μL of GL@Cy7 was dissolved in 3 mL PBS and placed in a dialysis bag. The dialysis membrane was then submerged in a vial containing 30 mL of PBS under constant agitation. At predetermined time intervals, a 100 μL aliquot of the dialysate was extracted and replaced with an equal volume of PBS 100 μL. The concentration of Cy7 released into the dialysate was quantified using UV–vis spectroscopy.

### Targeting efficiency of GL

To determine the targeting efficiency of GL *in vitro,* cells were treated with L@Cy7 or GL@Cy7 for 4 h. To investigate whether the transport of Gln-DL is dependent on the Gln-ASCT2 axis, cells were pre-incubated with Gln (4 mM), GPNA (3 mM), or ASCT2 antibody (1:500) at 37℃ for 4 h before addition of GL@Cy7. After that, cells were harvested, fixed, counterstained with DAPI, and the intracellular Cy7 signals in each group were detected by fluorescence microscopy (ZEISS Apotome 3).

To study the targeting efficiency of GL *in vivo*, 1x10^7^ cells were subcutaneously injected into the left groin of mouse to establish the subcutaneous tumor model. When the tumor volume reached around 100 mm^3^, GL@Cy7 and L@Cy7 were intravenously administered. The mice were imaged by an in vivo imaging system (Guangzhou Biolight Biotechnology Co., AniView100) to detect Cy7 signals at 1 h, 4 h, 8 h, 12 h after micelle intravenous administration. After that, tumors and major organs were dissected and collected for *ex vivo* fluorescence imaging.

### ROS detection

The intracellular ROS was detected by a DCFH-DA probe (Beyotime). Briefly, SiHa cells were seeded in 24-well plates at 5 × 10^4^ cells per well and treated with different nanoformulas. After that, cells were rinsed with PBS for three times and incubated with DCFH-DA (10 μM) in serum-free medium at 37 ℃ for 20 min. Post-staining, cells underwent three additional washes with serum-free medium to remove excess probe. ROS-dependent fluorescence was captured by fluorescence microscopy (ZEISS Apotome 3) with standardized parameters: 20 × objective, 488/509 nm excitation/emission, and consistent 204.27 ms exposure. Images were quantitatively analyzed using ImageJ (v1.48), with data representing three independent biological replicates.

### ATP determination, mitochondrial function assessment and NADP^+^/NAPDH detection

For ATP determination, Enhanced ATP Assay Kit (Beyotime, S0027) was used. Cells were pre-seeded in 6-well plates overnight and incubated with various treatments for 24 h. Post-treatment cell lysates were centrifuged to collect the supernatants. 100 μL ATP-detection solution was added to the test wells for 5 min, then 20 μL supernatants or standards were added to the test wells. Luminescence signal was monitored using a Cytation 5 microplate reader (BioTek).

To investigate the effect of the fabricated nanoformulas on mitochondrial function, cells were stained with potential-sensitive JC-1 dye using Enhanced Mitochondrial Membrane Potential Assay Kit (Beyotime, C2003S). Briefly, treated cells were incubated with 10 μg mL^−1^ JC-1 for 20 min at 37 °C, washed twice with JC-1 Staining Buffer, and imaged on a ZEISS Apotome 3 fluorescence microscope under standardized conditions. JC-1 monomeric forms were visualized at 488 nm excitation/509 nm emission (650 ms exposure), while aggregated forms were detected at 558 nm excitation/575 nm emission (100 ms exposure), with the characteristic red-to-green fluorescence shift indicating depolarization of mitochondrial membranes.

The intracellular level of NADPH/NADP^+^ was detected using NADP^+^/NADPH Assay Kit with WST-8 (Beyotime, S0179), according to the manufacturer's protocol. Cell lysates from 1 × 10^6^ cells were prepared in 200 μL extraction buffer, then incubated at 60 °C for 30 min to selectively degrade NADP^+^. The samples were subsequently treated with G6PDH working solution to enzymatically convert residual NADP^+^ to NADPH, followed by adding NADPH chromogen solution. The absorbance of the samples was then measured at 450 nm using a microplate reader (Cytation 5, BioTek). The formula is as follows: [NADP^+^] = [NADP_total_] − [NADPH]; [NADP^+^]/[NADPH] = ([NADP_total_] − [NADPH])/[NADPH].

### Fluorescence activated cell sorting (FACS) analysis

For apoptosis analysis, cells were stained with Annexin V-APC/7-AAD kit (MULTISCIENCES, AP105-60). Briefly, cells were centrifuged, washed with binding buffer, and resuspended to 1 × 10^7^ cells/mL. Then, 100 μL cell suspension was stained with 5 μL Annexin V-APC and 10 μl 7-AAD, incubated for 5 min in the dark, and analyzed by flow cytometry (BD, Celesta). The following gating strategy was detailed in Fig. S2a: debris exclusion via FSC/SSC (FSC threshold: 5,000), doublet exclusion using FSC-H/FSC-A, with fluorescence detection parameters for Annexin V-APC (λex = 640 nm, λem = 670 nm; voltage: 320, threshold: 1500) and 7AAD-PerCP-Cy5.5 (λex = 488 nm, λem = 685 nm; voltage: 320, threshold: 1370). Background fluorescence was defined by unstained controls, with positive populations established at > 99th percentile of control values. Spectral compensation between APC and PerCP-Cy5.5 was calculated using single-stained controls in FACSDiva.

For cell cycle analysis with PI (Elabscience, E-CK-A351), approximately 5 × 10^5^ cells are harvested, pelleted by centrifugation, washed with PBS, and fixed in 70 % ethanol (−20 °C) for 1 h. Fixed cells are washed, then treated with RNase A (1 mg/mL) at 37 °C for 30 min to ensure specific DNA staining. Cells are subsequently stained with PI (50 μg/mL) in the dark at 4 °C for 30 min to assess DNA content. Cells were gated via FSC/SSC (FSC threshold: 5,000) and doublets excluded using PE-A/PE-W dot plots (Fig. S2b). PI was detected in the PE channel (λex = 488 nm, λem = 575 nm; voltage: 295, threshold: 295) and analyzed using FlowJo's Cell Cycle platform without compensation.

To assess MRP2 substrate accumulation, cells were treated with various nanoformulations for 24 h and then incubated with 1 μM CDCFDA for 30 min. The cell size gate, based on FSC and SSC, was set up first to exclude cell debris and clumps (FSC threshold: 5,000) (Fig. S2c). The fluorescence of CDCFDA was detected in PE channel (λex = 488 nm, λem = 575 nm; voltage: 295, threshold: 295).

All BD Celesta acquisitions captured ≥ 10,000 events per sample analyzed in FlowJo v10.8.1, with data representing means from three biological replicates.

### Cell immunofluorescence staining

Cells undergoing different treatments were fixed in 4 % PFA for 15 min and blocked with Immunol Staining Blocking Buffer for 20 min. Next, cells were incubated with the primary antibody against HKⅡ (1:500, Immunoway, YM8118), ASCT2 (1:500, Proteintech, 20350–1-AP), CD44 (1:500, Immunoway, YM8279), MRP2 (1:500, Immunoway, YT2840), and γ-H2AX (Beyotime, C2035S) at 4 °C overnight and the goat anti-rabbit secondary antibody at room temperature for 1 h. At last, cells were counterstained with DAPI and visualized by fluorescence microscopy (ZEISS Apotome 3).

### *In vitro* hemolysis assay

The hemolytic activity of the nanoformulas was determined using red blood cells (RBC). GL@LND at various concentrations in ultrapure water was mixed with a 2 % RBC suspension and incubated at 37 ℃. After 2 h, the solution was centrifuged at 1200 rpm for 15 min, and the absorbance of the supernatant was measured at 540 nm through a microplate reader.

### Tissue histology

The cervical tissue microarray (HUteS30PG01-1) containing 15 paired cancer and *para*-tumor tissues was purchased from Shanghai Outdo Biotech Co., Ltd. Specifically, the tissue chip was dried, deparaffinized, and rehydrated following standard procedures. The sections were subjected to heat-induced antigen retrieval. Immunohistochemical staining was performed with anti-HKⅡ antibody (1:200,Immunoway, YM8118) or anti ASCT2 antibody (1:800, Proteintech, 20350–1-AP). Aipathwell (Wuhan Servicebio Technology Co., Ltd., China) was used to quantify the positive staining area.

For histological analyses of mice, animals were sacrificed, and the tumors and major organs were harvested for paraffin embedding. Sections of the major organs were stained with hematoxylin and eosin (H&E) to evaluate the abnormal cells and the morphology of tissues under an optical microscope. Concurrently, tumor sections were subjected to immunofluorescence analysis of Ki-67 (1:200, Servicebio, GB151499), 4-HNE (1:400, Servicebio, GB150073), 8-OhdG (1:200, Bioss, bs-1278r), and MRP2 (1:200, Immunoway YT2840), with imaging performed on a ZEISS Apotome 3 system. Additionally, terminal deoxynucleotidyl transferase (TdT) dUTP nick-end labeling (TUNEL) staining was conducted on tumor sections using a commercial kit (Servicebio, G1501) to detect apoptotic cells. The procedure involved sequential steps, including deparaffinization, antigen retrieval with Proteinase K, membrane permeabilization, reaction solution incubation, and nuclear counterstaining with DAPI. Fluorescent signals were imaged using a ZEISS Apotome 3system.

### Statistical analysis

Statistical analyses were performed using GraphPad Prism (v8.0.1), with data presented as mean ± SD. Significance levels were set at *P < 0.05, **P < 0.01, and ***P < 0.001.

### Animal treatment

To investigate the antitumor performance of the nanoformulas, DDP unresistant and resistant cervical carcinoma models were established. When the tumor sizes were up to ≈50 mm^3^, mice were intravenously administered saline, free LND, free DDP, GL, GL@LND, GL@DDP, GL@LND@DDP at LND and DDP doses of 10mg kg^−1^ and 2 mg kg^−1^, respectively. The administrations were conducted every two days for a total of eight doses. Tumor volumes and body weights were monitored in each group. After the mice were sacrificed, tumors, serums, and main organs of mice were harvested for further analysis.

## Results and discussion

**Fig. 2:**
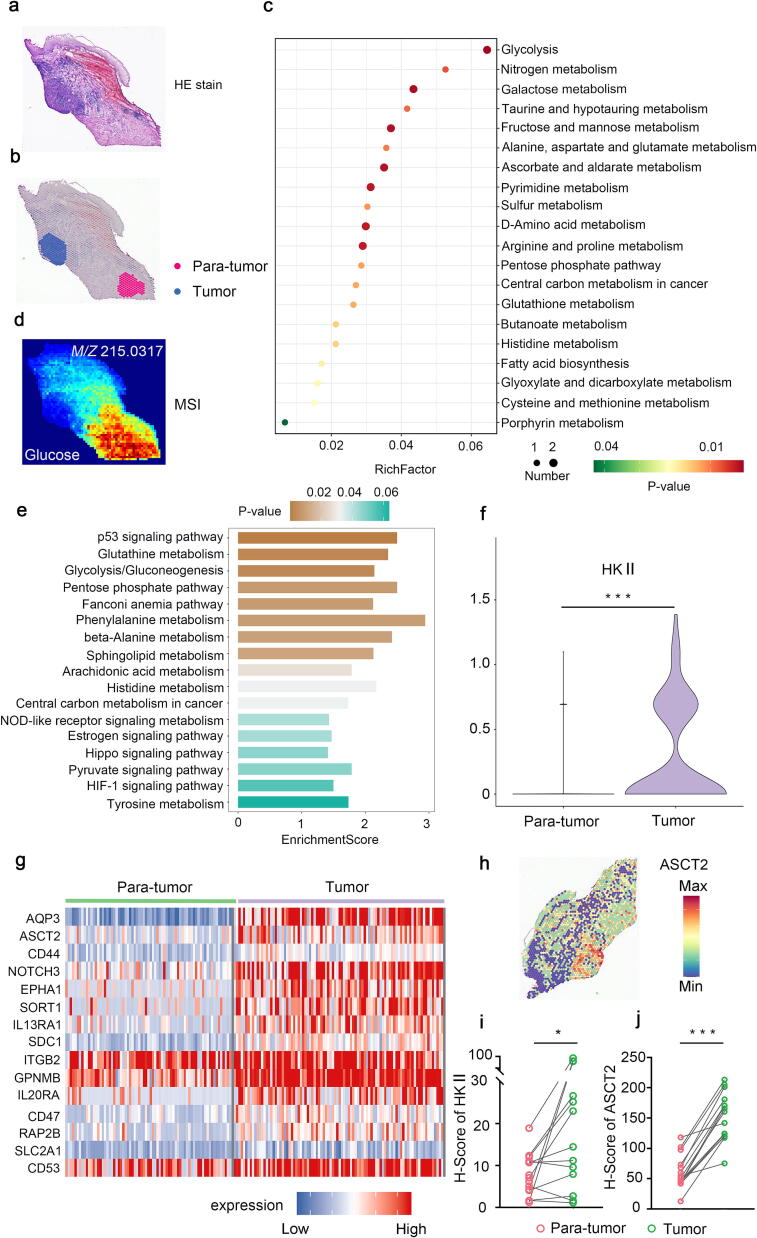
Spatial multi-omics analysis unveils that glycolysis is elevated and ASCT2 is overexpressed in the tumor regions of CC. a) H&E image of CC tissue section. b) Histological sections of CC in HE-stained images. c) Main up-regulated metabolic pathways in tumor regions identified through spatial transcriptomics. d) MS image of glucose (*m*/*z* 215.0317) in tumor regions and adjacent non-tumor regions. e) The top 15 KEGG pathways enriched in tumor regions identified through spatial transcriptomics. f) The transcriptional levels of HKⅡ in each group. Statistical significance was calculated via Student’s *t*-test, ***P＜0.001. g) Heatmap showing expression of cell membrane receptors that are highly present in tumor areas and not expressed on immune cells. h) Spatial transcriptomics feature plots of ASCT2. i-j) H-score quantification of HK2 (i) and ASCT2 (j) expression in cervical carcinoma versus paired adjacent tissues. Statistical significance was calculated via a paired Student’s *t*-test, *P＜0.05, ***P＜0.001.

For a start, histological sections of CC were examined and annotated by a pathologist, who identified tumor regions and adjacent non-tumor regions based on the morphological characteristics observed in hematoxylin and eosin (H&E)-stained images (Fig. 2, Fig. 2). Subsequent spatial transcriptomics analysis confirmed this partitioning, demonstrating that cells in H&E-annotated tumor regions primarily mapped to cluster 2, whereas those in adjacent non-tumor regions predominantly localized to cluster 0, thereby molecularly validating the initial histological annotations (Fig. S3a,b). This annotation was further validated by elevated expression of established carcinoma markers, including CDKN2A, TP63, MKI67, CCNB1, and TOP2A, specifically in the histologically-defined tumor regions (Fig. S4).

Spatial metabolomics via airflow-assisted desorption electrospray ionization mass spectrometry imaging (AFADESI-MSI) was specifically mapped to histopathologically defined tumor and non-tumor regions to validate glycolysis addiction in CC. Through partial least squares discriminant analysis (PLS-DA), region-specific discriminating metabolites were accurately extracted (Fig. S5). These metabolites were subsequently imported into the Kyoto Encyclopedia of Genes and Genomes (KEGG), confirming a significant upregulation of glycolysis in tumor regions compared to adjacent non-tumor areas (Fig. 2c). The microscopy-MSI overlay image corroborated that the levels of glucose, which are metabolized through glycolysis, were elevated in the tumor region of the tissue (Fig. 2d). Additionally, the in situ spatial transcriptomics analysis revealed significant enrichment of glycolysis pathway in tumor versus non-tumor regions (Fig. 2e), consistent with spatial metabolomics findings. These results suggest that targeting glycolysis could be an effective therapeutic strategy against CC.

Metabolic enzymes serve as crucial nodes in biological metabolic networks, making them established targets for metabolic reprogramming [10]. Consistent with prior reports [[17], [18], [19], [20], [21]], elevated expression of hexokinase II (HKⅡ), phosphofructokinase (PFKP), pyruvate kinase M (PKM), and lactate dehydrogenase A (LDHA) was observed in the tumor regions of CC compared to non-tumor regions (Fig. 2f, Fig. S6). Given HKⅡ's role in catalyzing the initial step of glycolysis [17], we prioritized its inhibition to disrupt glycolytic flux. LND is an effective inhibitor of HKⅡ, but it has low bioavailability and significant hepatotoxicity [22]. To improve the efficiency of the LND, we aimed to develop a liposomal delivery system for targeted CC delivery. Further spatial transcriptomics analysis identified ASCT2, a glutamine transporter, as substantially upregulated in tumors but absent on immune cells (Fig. 2g-h). Notably, ASCT2 showed higher expression differences than the established nanocarrier target CD44 [23] (Fig. 2g).

Multidimensional validation established HKⅡ/ASCT2 dysregulation as pivotal in CC pathogenesis. Reanalysis of a public single-cell RNA-seq dataset (GSE208653), comprising 3 cervical carcinoma and 2 normal cervical specimens, enabled identification of distinct epithelial subpopulations through cell typing (Fig. S7a-d), which demonstrated significant upregulation of glycolytic pathways alongside elevated HKⅡ and ASCT2 expression specifically within tumor epithelial cells compared to their normal counterparts (Fig. S7e-i). This cellular-resolution finding was reinforced by bulk transcriptomics in TCGA-CESC (306 tumors vs. 13 normals), demonstrating consistent HKⅡ/ASCT2 upregulation (Fig. S8a-b). Protein-level validation by immunohistochemistry using a 15-paired CC/adjacent tissue microarray confirmed this dysregulation, showing increased HKⅡ expression in tumors compared to adjacent tissue in 12 of 15 pairs and universal ASCT2 upregulation in all 15 tumors (Fig. 2i, Fig. S9-S10, Table S1). Western blot analysis of 12 paired cervical specimens also confirmed elevated HKⅡ expression in the majority of tumors (10/12) and universal ASCT2 overexpression (12/12) relative to matched normal tissues (Fig. S11, Table S2). Cross-species conservation was evidenced in SiHa-xenograft mice models (n = 5 tumor/normal pairs) and multiple CC cell lines (Fig. S12-16). Comparative CD44 expression analysis in the human single-cell RNA-seq dataset, TCGA cohort, mouse tissues and cellular systems supports the superior differential expression of ASCT2 (Fig. S7j, S8c, S13 and S16). This systematic cross-validation, spanning single-cell resolution, patient databases, clinical samples, animal models, and cellular systems, strongly reinforces the central finding of HKⅡ/ASCT2 upregulation as a hallmark of cervical cancer. Based on these findings, we developed glutamine-functionalized liposomes for targeted ASCT2-mediated delivery of LND.

**Fig. 3:**
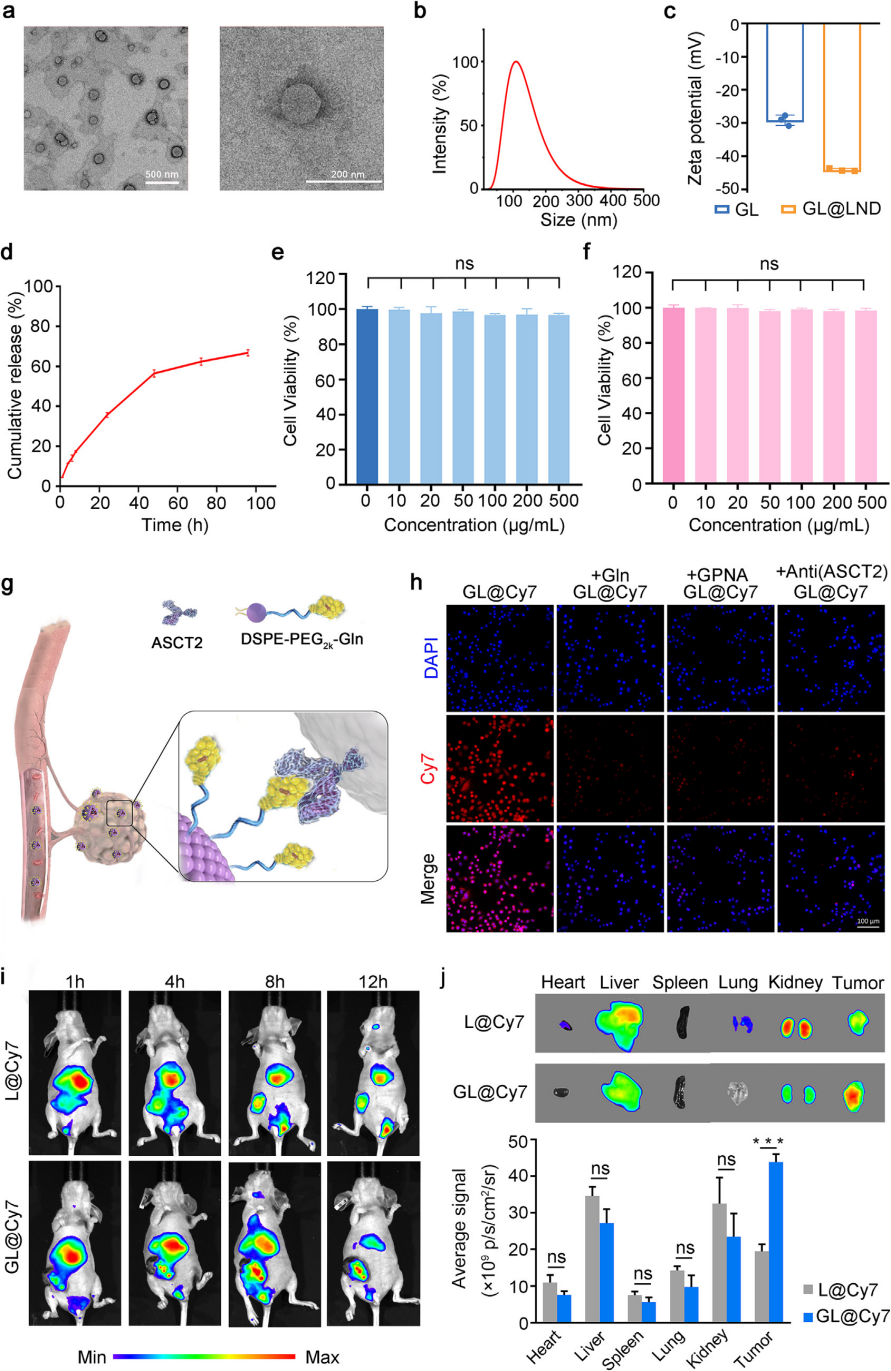
Physicochemical properties of the synthesized GL. a) Representative TEM images of GL@LND at low (left) and high (right) magnifications. b) Size distributions for GL@LND determined by DLS. c) Zeta potentials of GL and GL@LND (n = 3). d) The cumulative release of Cy7 from GL@Cy7. e-f) The cell viability of SiHa cells (e) and End1/E6E7 (f) cells was measured after incubation with GL at different concentrations for 48 h (n = 3). g) The scheme illustrates that CC specific GL uptake was achieved through Gln-ASCT2 recognition. h) Validation of the involvement of Gln-ASCT2 axis in the tumor-specific targeting of GL. Competitive binding assays were performed in SiHa cells pre-treated with excess free glutamine (Gln), the selective ASCT2 inhibitor GPNA or ASCT2 blocking antibody prior to incubation with GL@Cy7. i) Living imaging depicting the in vivo distribution of different formulations at different times. j) Ex vivo imaging and fluorescence quantification of dissected tumors and major organs harvested from tumor-bearing mice 12 h post-intravenous administration of L@Cy7 or GL@Cy7 (n = 3). Statistical significance was calculated via one-way ANOVA, ns means no significant, ***P＜0.001.

PEG_2k_-DSPE has been utilized to create long-circulating liposomes, which demonstrated superior stability and drug entrapment efficiency compared to conventional formulations [[24], [25], [26]]. The grafting of Gln onto PEG_2k_-DSPE was confirmed by ^1H^ NMR spectroscopy and Matrix-Assisted Laser Desorption/Ionization Time of Flight Mass Spectrometry (MALDI-TOF-MS) analysis (Fig. S17-18). GLs were formulated using soya phosphatidyl choline (SPC), cholesterol, and Gln-PEG2k-DSPE. Transmission electron microscopy (TEM) and dynamic light scattering (DLS) analyses further characterized the GLs as spherical nanoparticles (NPs) with an average diameter of 108 nm and a zeta potential of −44.18 mV (Fig. 3a-c). The entrapment and loading efficiency of GL were found to be 94 ± 2.54 % and 7.5 ± 0.23 %, respectively. To evaluate the drug release kinetics of GL, sulfo-Cyanine7 (Cy7) was utilized as a model compound and encapsulated in GL. Over an extended period, GL@Cy7 demonstrated a gradual release of the model compound, indicating the sustained release capability of GL (Fig. 3d). We then investigated the biocompatibility of GL using the human cervical epithelial cell line End1/E6E7 and cervical cancer cell line SiHa. At GL concentrations up to 500 µg mL^−1^, no cytotoxic effects were detected, indicating that GL does not exhibit general cytotoxicity within this range (Fig. 3e-f).

We examined the tumoral targeting effect of GL (Fig. 3g). As the results shown, the grafting of Gln significantly enhanced the accumulation of Cy7 in SiHa cells compared to the unmodified liposomes (Fig. S19). To further validate the involvement of Gln-ASCT2 axis in the tumor-specific targeting of GL, SiHa cells were pre-incubated with excessive Gln molecules, GPNA (an ASCT2 antagonist) or an ASCT2 antibody. Notably, the fluorescence of Cy7 was substantially diminished following pretreatment with Gln, GPNA, or the ASCT2 antibody, confirming that the high targeting efficiency of GL is primarily dependent on the Gln-ASCT2 interaction (Fig. 3h, S20). To support this notion *in vivo*, SiHa tumor-bearing xenografts were established. Compared to L@Cy7, the fluorescence of Cy7 was mainly gathered in the tumor region at 12  h post intravenous administration of GL@Cy7 (Fig. 3i). Consistent observations were made through *ex vivo* bioluminescence analysis of the excised tumors and major organs (Fig. 3j). Overall, these findings confirm the high tumor-targeting feasibility of GL.

**Fig. 4:**
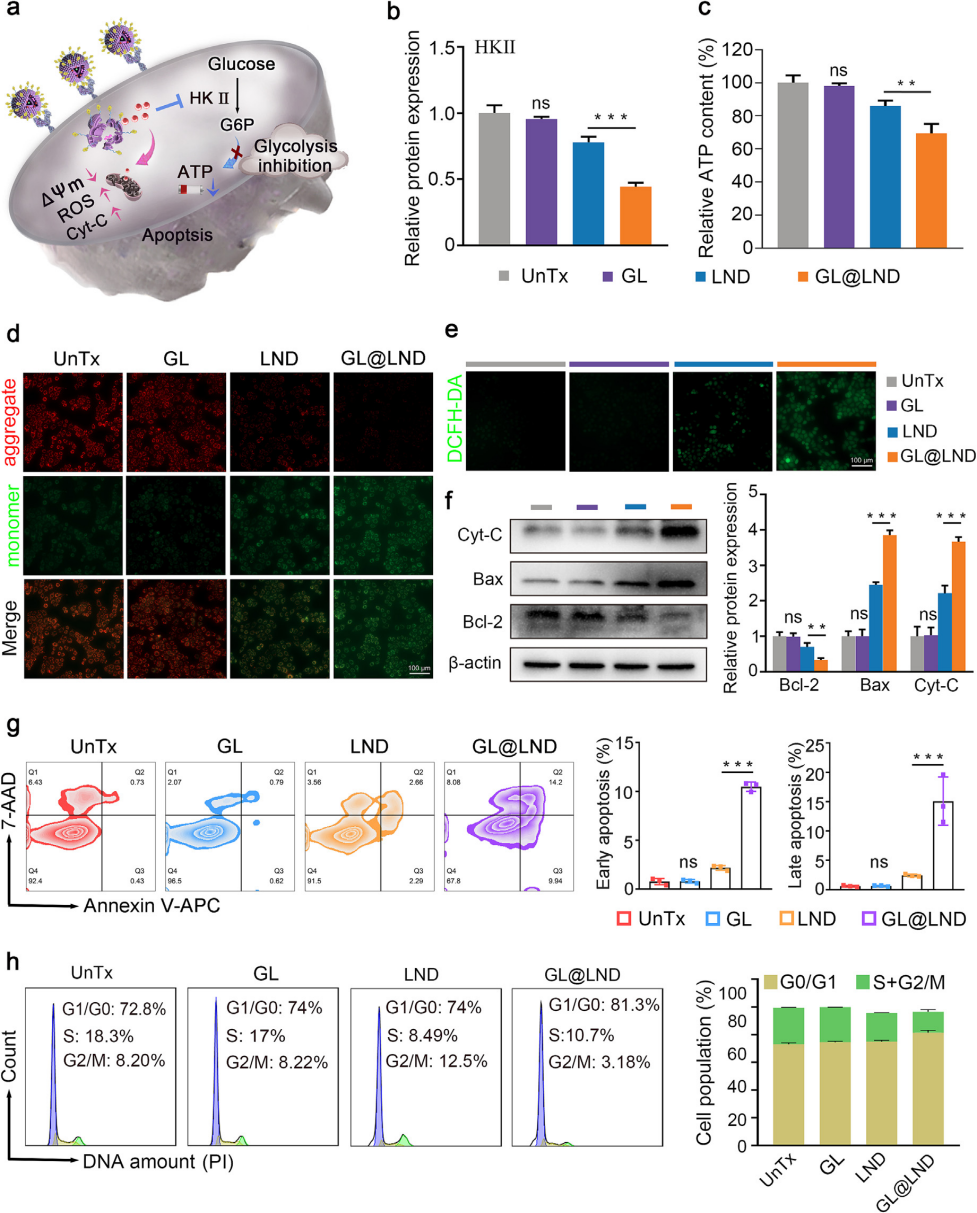
GL@LND for glycolysis reprogramming. a) Schematic of the mechanisms of GL@LND-mediated cell apoptosis. GL@LND markedly down-regulated glycolysis by inhibiting HKⅡ, triggered the dissipation of mitochondrial transmembrane potential, and facilitated reactive oxygen species (ROS) generation, thereby cutting off the energy supply and causing cellular damage for tumor regression. b) The relative protein levels of HKⅡ in the untreated group (UnTx), the GL group, the free LND group, and the GL@LND group. c) The content of intracellular ATP in each group. d) Representative fluorescence images of JC-1 probe to measure mitochondrial membrane depolarization in SiHa cells after various treatments. Aggregated JC-1 complexes (red fluorescence) represent healthy mitochondria, while monomeric dye dispersion (green fluorescence) indicates membrane depolarization. Scale bar: 100 μm. e) Representative DCFH-DA stained images of SiHa cells from different treatment groups. Scale bar: 100 μm. f) Western blot analysis of the protein levels of Cyt-C, pro-apoptotic Bax, and anti-apoptotic Bcl-2 in each group. g) The percentages of early apoptosis (annexin V^+^7-AAD^−^) and late apoptosis (annexin V^+^7-AAD^+^) in SiHa cells from each group were analyzed by flow cytometry. h) FACS analysis of the effects of different formulas on cell cycle in SiHa cells. Data are represented as mean ± SD (n = 3). Statistical significance was calculated via one-way ANOVA, ns means not significant, **P＜0.01, ***P＜0.001.

Next, we assessed whether the synthesized NPs can reprogram glycolysis *in vitro* (Fig. 4a). As the results shown, GL had a negligible effect on the expression of HKⅡ in SiHa cells. In contrast, GL@LND dramatically inhibited the expression of HKⅡ, much more effective than the function of free LND, verifying the high targeting and release efficiency of GL (Fig. 4b, S21). Considering that glycolysis serves as the primary pathway for ATP production in tumor cells, we measured the intracellular ATP levels across different groups. A substantial reduction in ATP levels was observed in the GL@LND group (Fig. 4c). This reduction depended on the delivery of LND, as treatment with unloaded GL did not affect ATP levels. Notably, we noted that free LND only decreased ATP content by 14.03 %, validating the advantages of GL in delivering LND (Fig. 4c). Together, these pieces of evidence validate that delivery of LND by GL effectively suppresses the glycolysis pathway and restricts the energy supply in tumor cells.

LND has been shown to induce the destruction of mitochondrial structure [[27], [28], [29]]. We thus monitored the damage of mitochondria by using JC-1, which is known to accumulate in mitochondria in a potential-dependent manner. Under high membrane potential, JC-1 forms red J-aggregates, whereas in mitochondria with low membrane potential, it forms green J-monomers [30,31]. In comparison to free LND, GL@LND significantly decreased JC-1 aggregates and increased JC-1 monomers in SiHa cells, indicating the dissipation of mitochondrial membrane potential (Δψm) (Fig. 4d, S22). Since the generation of ROS is closely related to the Δψm alterations, we assessed the intracellular ROS levels using dichloro-dihydro-fluorescein diacetate (DCFH-DA), a widely utilized fluorescent probe for ROS detection in various biological systems. As the results shown, there was no observable difference in the fluorescence intensity of DCFH-DA between the PBS and GL groups. In contrast, GL@LND resulted in a ∼ 3.4-fold increase in the fluorescence of DCFH-DA compared to free LND, indicating a substantial induction of ROS (Fig. 4e, S23).

The depolarization of Δψm triggers the release of cytochrome *C* (Cyt-C), which subsequently activates the caspase-related apoptosis pathway and finally kills cancer cells. To support this notion, the apoptosis-related proteins were analyzed by Western blot. The expression levels of Cyt-C remained unchanged in the PBS and GL groups. However, the LND released from GL increased the expression of Cyt-C, which was more effective than free LND. Similar results were found in the expression of pro-apoptotic protein Bax. Conversely, the expression of anti-apoptotic protein Bcl-2 was minimized by the treatments of GL@LND (Fig. 4f). We further verified the pro-apoptotic effect of GL@LND by Annexin V-APC/7-AAD assays. Remarkably, GL@LND group induced 10.48 % early apoptosis and 15.10 % late apoptosis, which were 4.81-fold and 6.24-fold higher than those in the free LND group, verifying the enhanced efficiency of LND delivery by GL (Fig. 4g). Additionally, unlike the minor changes in the cell cycle seen with free LND, GL@LND treatment significantly increased the percentage of cells in the G0/G1 phases while markedly decreasing those in the S and G2/M phases. This indicates that the delivery of LND by GL maximally arrests SiHa cells in the G0/G1 phase of the cell cycle and prevents their entry into the S and G2/M phases (Fig. 4h). Together, the above cell-based assay confirmed that GL@LND significantly impedes glycolysis, induces Δψm depolarization, and promotes ROS generation, ultimately disrupting the energy supply and inducing cellular apoptosis.

**Fig. 5:**
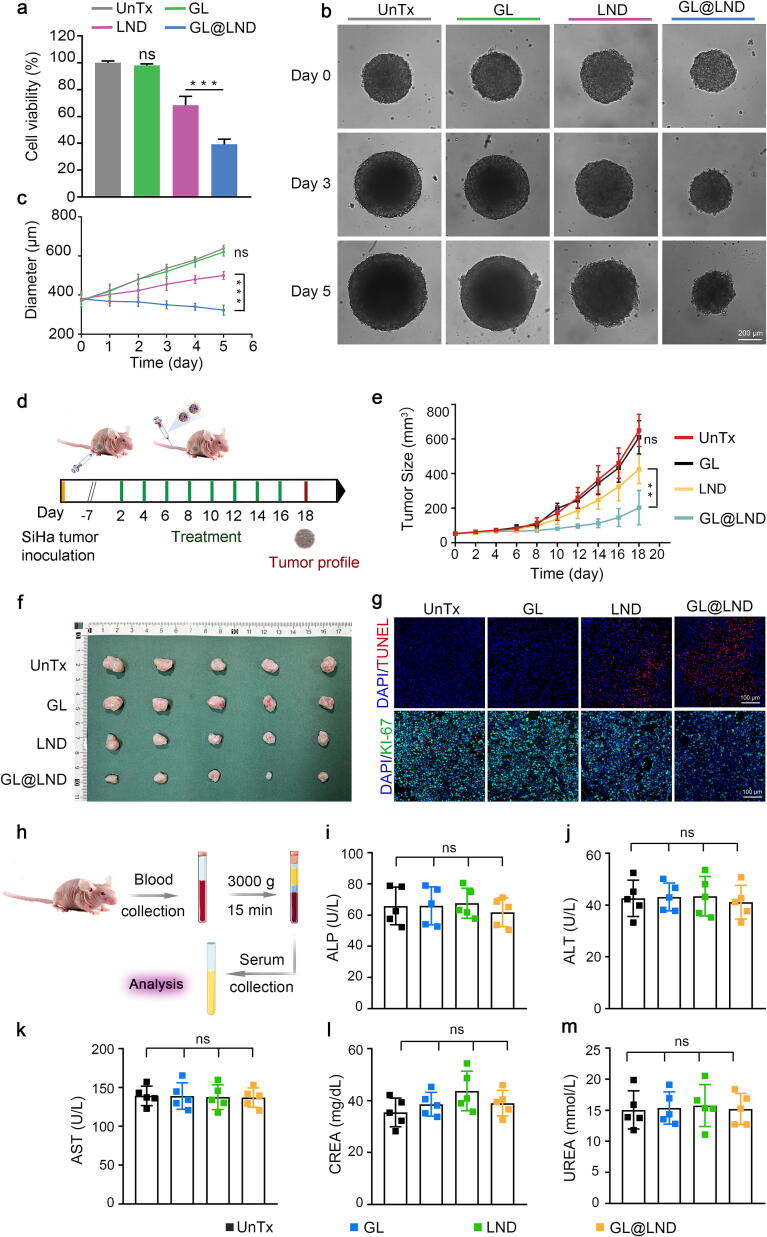
Evaluation of antitumor effect of GL@LND. a) Cell viability of SiHa cells after treatment with GL, free LND or GL@LND for 48 h. b) Representative images of SiHa MCTSs after incubation with various formulations at the different times. Scarle bars: 200 μm. c) The diameter changes in SiHa MCTSs in each group (n = 3). d) Schematic illustration of in vivo experimental design. Mice were intravenously administered saline, free LND, unloaded GL or GL@LND at an LND dose of 10 mg/kg. e) Average tumor growth curves displayed the tumor volume and tumor growth rate (n = 5). f) Photograph of excised tumors from mice after various treatments on day 18. g) TUNEL and Ki-67 image of tumor slices of mice receiving different treatments. Scale bar: 100 μm (n = 5). h) Diagrammatic representation of serum biochemical index analysis process. i-m) Serum biochemical indexes of ALP (i), ALT (j), AST (k), CREA (l) and UREA (m) in each group. Data are represented as mean ± SD (n = 5). Statistical significance was calculated via one-way ANOVA, ns means not significant, *P＜0.01, ***P＜0.001.

We following investigated the cytotoxicity of the nanoformulus on SiHa cells. Free LND induced a 31.50 % reduction in cell viability, whereas GL@LND resulted in a 60.72 % decrease in cell viability after 48 h incubation, confirming that GL enhances the bioavailability of LND (Fig. 5a, S24). To further assess the anti-proliferative effects of GL@LND, we established 3D multicellular tumor spheroids (MCTSs), which closely mimic drug efficacy in solid tumors. The diameters of SiHa MCTSs grew nearly the same after PBS and unloaded GL treatment. In contrast, GL@LND treatment reduced the diameter to 350 μm after 5 days, a significant reduction compared to the free LND-treated group (Fig. 5b-c).

Encouraged by the efficacy of GL@LND *in vitro*, we investigated the performance of the fabricated nanoformulas in a SiHa cervical cancer mouse model. Before this, hemolysis test results indicated that GL had a negligible effect on erythrocytes (Fig. S25). The mice were assigned to five groups and injected intravenously with saline, GL, free LND or GL@LND every two days for a total of eight doses (Fig. 5d). The GL group showed tumor growth rates comparable to the saline group, indicating the negligible effect of the empty carrier on CC growth. Notably, free LND showed a mild effect on tumor growth, whereas GL@LND resulted in a reduction of average tumor size by 68.77 %, indicating the superior efficacy of GL@LND in suppressing CC (Fig. 5e-f, S26).

Mechanistically, GL@LND potently inhibited glycolysis, evidenced by downregulated HKⅡ protein and reduced ATP levels in tumors (Fig. S27-28). This metabolic disruption was accompanied by elevated oxidative stress markers, including 4-hydroxynonenal (4-HNE, an indicator of lipid peroxidation) and 8-hydroxyguanosine (8-OHdG, a product of DNA and RNA oxidation) (Fig. S29). Mitochondrial damage was manifested through elevated cytochrome *C* release, while apoptosis was robustly confirmed via decreased Bcl-2 and increased Bax expression (Fig. S30). Immunofluorescence validated these findings, showing reduced Ki-67 proliferation marker and increased TUNEL apoptosis staining in the GL@LND group (Fig. 5g, S31). Importantly, GL@LND did not elicit systemic toxicity in mice, as evidenced by stable body weight (Fig. S32), unaltered liver parameters (ALT, ALP, AST) and kidney parameters (CREA and UREA) (Fig. 5h-m), as well as uncompromised tissue morphology and cellular structure in major organs (Fig. S33). Taken together, these data suggest that GL@LND exhibits a superior effect in inhibiting CC development.

In addition to energy supply, glycolysis has also been documented to play important roles in chemoresistance [[32], [33], [34], [35]]. DDP-induced chemoresistance has been widely reported in the clinical treatment of CC. The accumulation of DDP in cells and the induction of DNA damage are the two critical steps in its mechanism of action. However, on one hand, glycolysis-generated ATP glycolysis-generated ATP can promote MRP2 expression, which facilitates DDP efflux and reduces DDP intracellular accumulation [7,16,[36], [37], [38]]. On the other hand, R5P, an intermediate in the pentose phosphate pathway (PPP) branch of glycolysis, is crucial for DNA biosynthesis to repair DNA damage caused by DDP [5,9,10,39]. Thus, inhibition of glycolysis may induce the chemoresistant-to-chemosensitive transition by decreasing MRP2-mediated DDP efflux and suppressing R5P-mediated DNA repair.

**Fig. 6:**
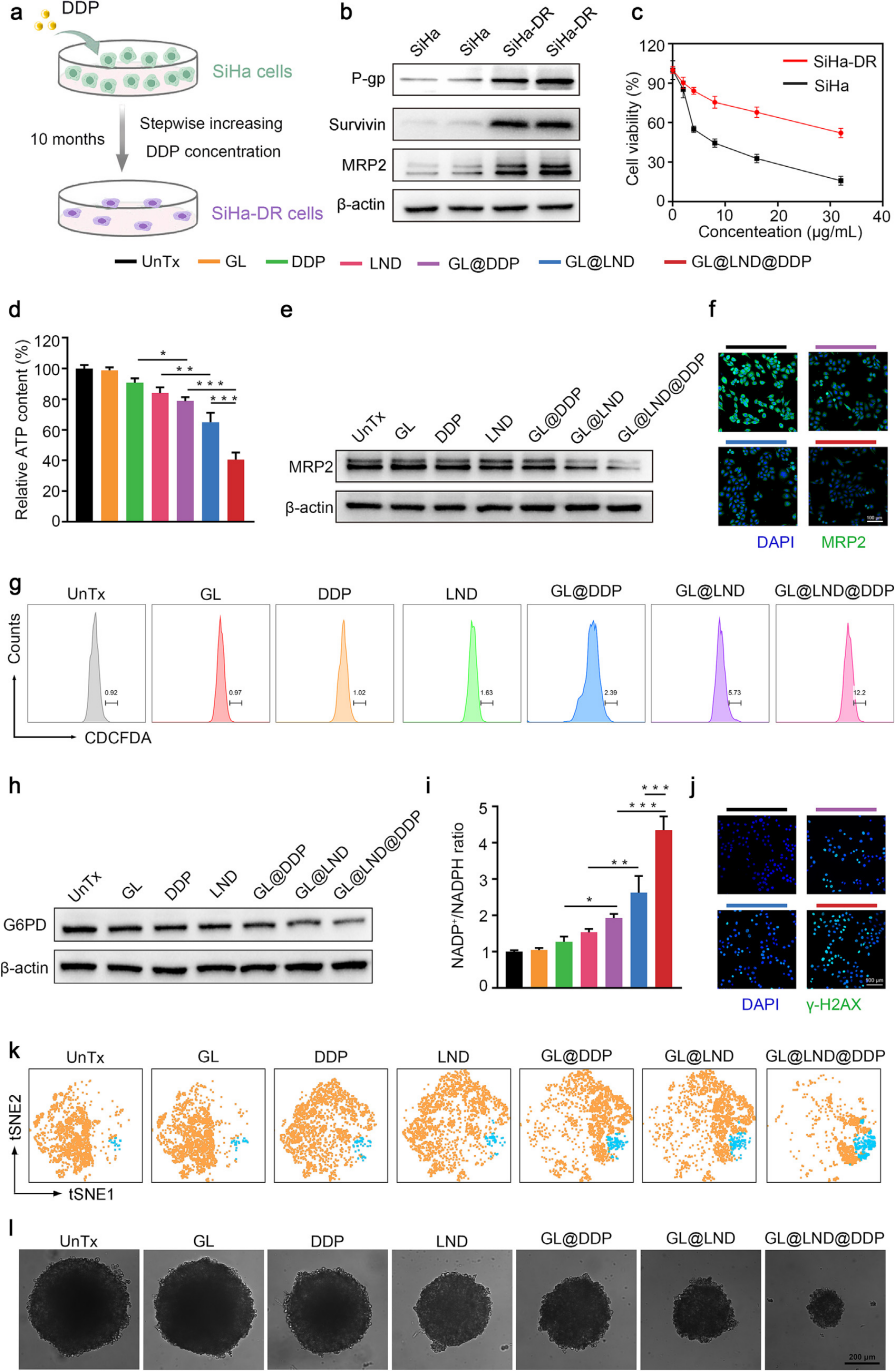
GL@LND@DDP reverses chemoresistance *in vitro*. a) Schematic illustration of the establishment of SiHa-DR cells by stepwise increasing DDP concentration and intermittent administration for 10 months. b) The expression of drug resistance-associated proteins in each group. c) Dose-response curves showed that SiHa-DR cells exhibited resistance to DDP compared to SiHa cells. d) Intracellular ATP content in SiHa-DR cells in different groups. e) Western blot analysis of the expression of MRP2 in each group. f) Immunoblotting analysis of MRP2 protein across different groups. Scale bars: 100 μm. Refer to Fig. S41 for complete data. g) Flow cytometry analysis of CDCFDA accumulation after indicated treatment. h) The protein levels of G6PD in different groups. i) NADP^+^/NADPH ratios in SiHa-DR cells subjected to various treatments. j) Representative fluorescence images of γ-H2AX in SiHa-DR cells. Refer to Fig. S43 for complete data. k) The t-SNE maps of apoptosis cells labeled in blue. l) Representative images of SiHa-DR MCTSs after indicated treatment. Refer to Fig. S48 for complete data. All data are represented as mean ± SD (n = 3). Statistical significance was calculated via one-way ANOVA, *P＜0.05, **P＜0.01, ***P＜0.001.

To substantiate this hypothesis, a DDP-resistant SiHa cell line (SiHa-DR) was established, as demonstrated by western blot analysis and CCK8 assay results (Fig. 6a-c). The multidrug resistance (MDR) proteins, including MRP2 and P-glycoprotein (P-gp), exhibited elevated expression levels in SiHa-DR cells compared to SiHa cells. A similar trend was observed in the expression of Survivin, which is closely linked to MDR protein expression (Fig. 6b, S34). Furthermore, growth curve analysis conducted by CCK8 assay and IC50 determination confirmed the successful induction of SiHa-DR (Fig. 6c). Notably, the chemoresistant phenotype did not alter the expression of HKⅡ and ASCT2 in SiHa cells (Fig. S35), and the physicochemical properties and targeting efficiency of GL-derived nanoformulas remained unaltered in the context of SiHa-DR cells (Fig. S36-39).

We subsequently examined the impact of the synthesized NPs on overcoming chemoresistance. In alignment with the previously mentioned findings, the incorporation of LND resulted in a significant reduction in cellular ATP levels (Fig. 6d). Western blot analysis revealed that the expression of ATP-dependent MRP2 in the GL@LND group decreased by 35.45 % compared to the PBS group and by 25.26 % compared to the free LND group. Notably, the most significant reduction in MRP2 expression was observed with GL@LND@DDP at 70.45 %, followed by GL@LND at 35.45 %, and GL@DDP at 19.97 % (Fig. 6e, S40). This emphasizes the synergistic effects of the combination strategy. Similar results were found in the immunofluorescence staining of MRP2 (Fig. 6f, S41). Additionally, FACS analysis indicated a significant accumulation of CDCFDA, a substrate of MRP2, in the GL@LND group. The combined delivery of LND and DDP by GL resulted in the highest cellular accumulation of CDCFDA, reinforcing the synergistic interaction between these two components in GL (Fig. 6g). These results demonstrated the effectiveness of GL in delivering LND to suppress DDP efflux in SiHa-DR cells.

To further explore the potential of GL-mediated delivery of LND in suppressing R5P-induced DNA repair, we examined markers associated with the PPP signaling pathway. Glucose-6-phosphate dehydrogenase (G6PD) is the initial and rate-limiting enzyme of the PPP. It mediates the production of ribose-5-phosphate (R5P) and the conversion of NADP^+^ to NADPH. Western blot analysis revealed that GL@LND significantly reduced G6PD expression compared to the control group. Correspondingly, the NADP^+^/NADPH ratio in SiHa-DR cells was markedly increased. Importantly, the lowest G6PD expression and the highest NADP^+^/NADPH ratio were observed in the GL@LND@DDP group, indicating a synergistic effect of the co-delivery of LND and DDP via GL (Fig. 6h-i, S42). These findings suggest that the inhibition of glycolysis by GL@LND significantly impairs PPP signaling activity, thereby impeding R5P-mediated DNA repair in SiHa-DR cells. To further substantiate this hypothesis, we conducted γ-H2AX staining to identify DNA damage across different groups. Compared to GL@DDP, GL@LND@DDP resulted in a 2.67-fold increase in DNA damage in SiHa-DR cells (Fig. 6j, S43). Additionally, the Δψm loss, ROS generation, and apoptosis rate occupied the highest level in the GL@LND@DDP group (Fig. S44-46, Fig. 6k). These promising results indicated that a combination tactic of LND and DDP gave a great chance to rehabilitate chemosensitivity in CC.

We then explored the antitumor effect of the nanoformulas in SiHa-DR cells. As shown in treatment with free DDP alone hardly reduced cell viability, while encapsulation of DDP by GL blunted cell viability by 38.70 %, confirming that GL enhances the bioavailability of DDP. Notably, fabricated GL@LND@DDP caused ∼ 86.70 % lethality, indicating that the introduction of LND effectively reverses DDP-induced chemoresistance (Fig. S47). Similar results were observed in MCTSs. The GL@DDP only showed mild differences in the diameters of MCTSs compared to PBS group. However, GL@LND@DDP dramatically decreased the diameters of MCTSs, suggesting that the incorporation of LND restores chemosensitivity in CC (Fig. 6l, S48).

**Fig. 7:**
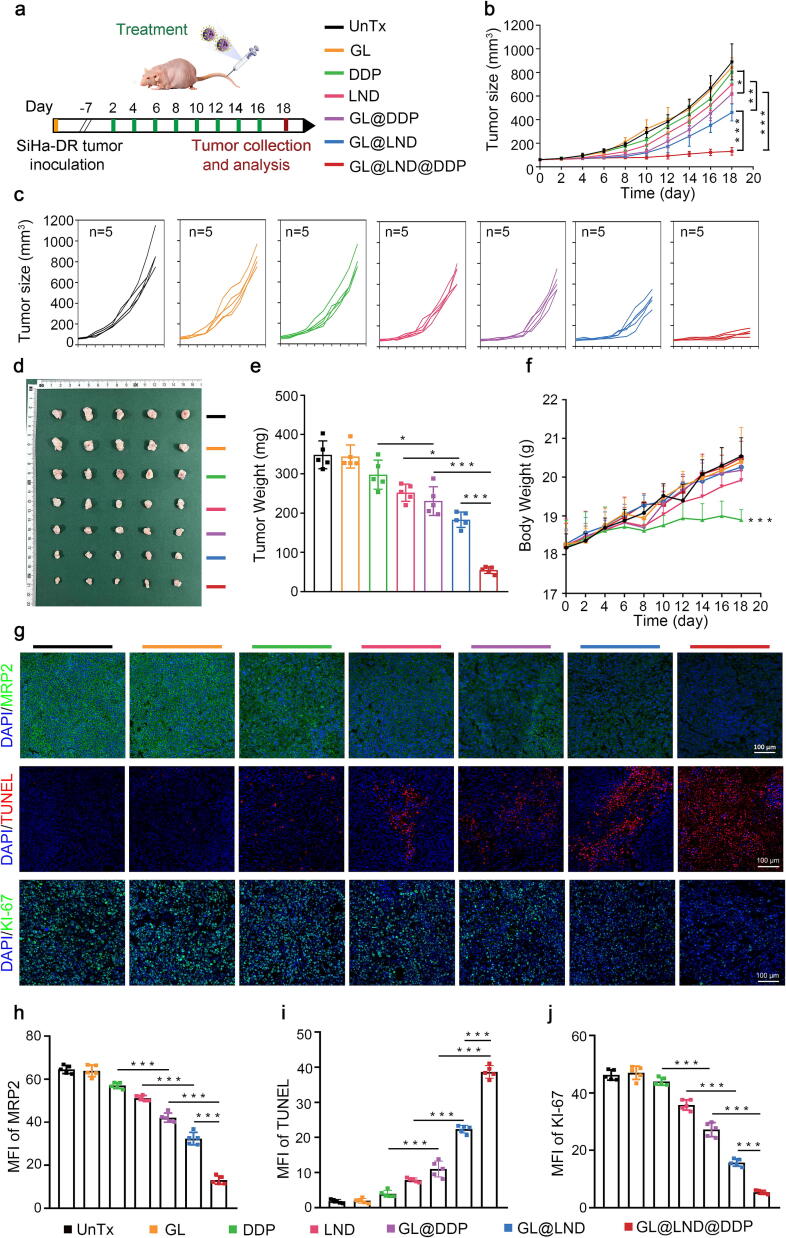
The *in vivo* anti-tumor action of GL@LND@DDP against chemoresistant cervical cancer. a) Schematic outline of experimental design for therapy. b) Average tumor growth curves. c) Individual tumor growth of SiHa-DR-tumor-bearing mice in different treatment groups. d) The images of tumors on day 18. e) Excised tumor weight following various treatments. f) Alterations in the body weight of mice throughout the treatment. g) Representative immunofluorescence images of MRP2, TUNEL and Ki-67 in tumor tissues from mice receiving various treatments. All data are represented as mean ± SD (n = 5). Statistical significance was calculated via one-way ANOVA, *P＜0.05, **P＜0.01, ***P＜0.001.

We further validated the effects in SiHa-DR xenograft model (Fig. 7a). There was a rapid and progressive growth of tumors in the saline and DDP groups, confirming that this model was resistant to DDP. Treatment with GL@LND@DDP displayed a noticeable effect against tumor progression and reduced the mean tumor volume by 85.21 %. As repeated treatment with GL@DDP only reduced the average tumor size by 30.37 %, this demonstrates that the introduction of LND renders tumors susceptible to chemotherapy (Fig. 7b-e). Similar results were observed in the immunofluorescence analysis, in which the proliferation marker Ki67 occupied the lowest fraction while TUNEL showed the most significant cell apoptosis extent in the GL@LND@DDP group (Fig. 7f). The substantial decrease of MRP2 was also confirmed in the tumors of the GL@LND@DDP group. Moreover, unlike DDP-induced weight loss in nude mice, no weight alteration was observed DDP loaded groups (Fig. 7g). Also, no anomalous liver and kidney indices were observed in any of the groups, nor was there any histological damage to the main organs (Fig. S49-50). Together, these data demonstrate that targeting dysregulated glucose metabolism can rehabilitate chemosensitivity in CC.

## Conclusion

The failure of CC treatment is mainly due to significant side effects and the development of chemoresistance. Targeting metabolic vulnerabilities has emerged as a promising strategy to suppress cancer progression. Several researchers have proposed that glycolysis increases in CC. However, clinical translation faces two barriers: inadequate spatial resolution of glycolytic dependency mapping in clinical specimens, and unavailability of tumor-selective nanovectors for bioavailable delivery of the hepatotoxic glycolysis inhibitor LND.

To validate this metabolic vulnerability in CC, we present an integration of mass spectrometry imaging-based spatial metabolomics and microarray-based spatial transcriptomics to validate the glycolysis addiction in the same cervical cancer sample. Glycolysis pathway was validated to be upregulated in tumor regions compared to adjacent non-tumor areas in CC. Additionally, ASCT2, a glutamine transporter, was identified as a superior CC-specific surface marker compared to the pan-cancer nanocarrier targets like CD44. These findings were corroborated through multi-platform validation spanning single-cell RNA-seq dataset, TCGA cohorts, paired patient specimens, matched murine samples, and multiple CC cell lines.

Consequently, we developed glutamine-functionalized liposomes incorporating the glycolysis inhibitor LND to suppress the progression of CC. The incorporation of LND into GL enormously attenuated the growth of cervical cancer by cutting off ATP supply and inducing ROS-mediated cellular damage. More importantly, the introduction of LND significantly reversed DDP-induced chemoresistance in cervical cancer. On one hand, it suppressed MRP2-induced DDP efflux; on the other hand, it disrupted R5p-mediated DNA repair. As a result, the combination of LND and DDP exhibited synergistic effects in suppressing tumor growth in a DDP-resistant cervical cancer model. As the association between glycolysis and chemoresistance has been documented in various other tumor types, including breast, ovarian, and colorectal cancers [10,32,40,41], this observation suggests that targeting glycolysis to restore chemosensitivity, in combination with chemotherapy, may represent a generalized strategy for the treatment of chemoresistant cancers.

## Compliance with ethics requirements

All procedures performed in studies involving human participants were in accordance with the ethical standards of the Ethics Committee of the First Affiliated Hospital of Xinjiang Medical University (Approval NO.20220304–01), and the volunteer was fully informed and consented with the experiment. All animal studies were approved by the Ethics Committee of Animal Experimental Center of Xinjiang Medical University (Approval No. IACUC–20211016–62) and were carried out in compliance with all relevant ethical regulations.

## CRediT authorship contribution statement

**Xiaojiao Li:** Conceptualization, Investigation, Visualization, Resources, Writing – review & editing, Funding acquisition, Supervision. **Xiangchuan Qin:** Writing – original draft, Data curation, Formal analysis, Visualization, Software, Validation, Methodology, Investigation. **Xiejun Zhao:** Software, Validation, Formal analysis, Investigation, Methodology. **Yufeng Liu:** Methodology, Software, Validation, Formal analysis. **Ling Wang:** Data curation, Validation, Software. **Kefeng Li:** Investigation, Methodology. **Jinqiu Li:** Software. **Jia Ma:** Supervision, Visualization, Project administration. **Liangliang Dai:** Writing – review & editing, Supervision. **Ayshamgul Hasim:** Conceptualization, Funding acquisition, Project administration, Writing – review & editing.

## Declaration of competing interest

The authors declare that they have no known competing financial interests or personal relationships that could have appeared to influence the work reported in this paper.

## Data Availability

The raw transcriptomics data have been deposited in the SRA database under accession number PRJNA1307636. All other data that support the findings of this study are available from the corresponding authors upon reasonable request.
