# Supplementary material for: Spatial multi-omics guides ASCT2-targeted delivery of glycolysis inhibitor for cervical cancer suppression and chemoresistance reversal
Source: J Adv Res. 2025 Nov 16;86:655–70. doi: 10.1016/j.jare.2025.11.035 (PMC13453967; doi:10.1016/j.jare.2025.11.035)
Supplement: Supplementary file 1 [file mmc1.docx]

**Spatial Multi-Omics Guides ASCT2-Targeted Delivery of Glycolysis Inhibitor for Cervical Cancer Suppression and Chemoresistance Reversal**

*Xiaojiao Li^a,b,c,†^, Xiangchuan Qin*^a^*^, †^, Xiejun Zhao*^d^*, Yufeng Liu^b,c^, Ling Wang*^a^*, Kefeng Li^e^, Jinqiu Li*^a^*, Jia Ma*^d,f^*^*^, Liangliang Dai*^g^*^*^, Ayshamgul Hasim^a^^[1]^*

^a^ Department of Basic Medicine, Xinjiang Medical University and Xinjiang Key Laboratory of Molecular Biology of Endemic Diseases, Urumqi, China.

^b^ BioBank, The First Affiliated Hospital of Xi'an Jiaotong University, Xi'an 710061, Shaanxi, PR China.

^c^ Shaanxi Engineering Research Center for Biobank and Advanced Medical Research, The First Affiliated Hospital of Xi'an Jiaotong University, Xi'an 710061, Shaanxi, PR China.

^d^ School of Pharmacy, Health Science Center, Xi’an Jiaotong University, Xi'an 710061, Shaanxi, PR China.

^e^ College of Food Engineering and Nutritional Science, Shaanxi Normal University, Shaanxi, 620 West Chang’an Avenue, Xi’an 710119, Shaanxi, PR China.

^f^ Department of Medical Oncology, The First Affiliated Hospital, Xi'an Jiaotong University, Xi'an 710061, Shaanxi, PR China.

^g^ Xi'an Key Laboratory of Stem Cell and Regenerative Medicine, Institute of Medical Research, Northwestern Polytechnical University, Xi'an 710072, Shaanxi, PR China.

*Corresponding author: [mj2024@xjtu.edu.cn](mailto:mj2024@xjtu.edu.cn) (Jia Ma); liangliangdai@nwpu.edu.cn (Liangliang Dai); axiangu@xjmu.edu.cn (Ayshamgul Hasim)

**Keywords:** Spatial multi-omics analysis; Glycolysis; Cervical Cancer; Chemoresistance Reversal

**Figure S1.** Quality control metrics for spatial transcriptomics spots. The distributions of UMI counts (a), gene counts (b), and mitochondrial gene expression percentages (c) per spot are presented.

**Figure S2.** Gating strategy for detection of apoptotic cells (a), cell cycle (b), and CDCFDA accumulation (c).

**Figure S3.** Integrated single-cell clustering and spatial mapping of tumor microenvironment. (a) The UMAP image shows 9 identified clusters. (b) Spatial cluster distribution in tissue. H&E-annotated tumor regions primarily mapped to cluster 2, whereas those in adjacent non-tumor regions predominantly localized to cluster 0.

**Figure S4.** Expression of established carcinoma markers (CDKN2A, TP63, MKI67, CCNB1, and TOP2A) in histologically-defined tumor and non-tumor regions.

**Figure S5.** PLS-DA analysis of different regions in CC sample.

**Figure S6**. Spatial expression profiles of glycolytic enzymes PFKP, PKM and LDHA. Statistical significance was calculated via Student’s t-test, ****P*＜0.001.

**Figure S7.** Analysis of cervical cancer single-cell RNA-seq data (GSE208653). (a) UMAP visualization of single-cell distribution across samples after downscaling. (b) Unsupervised clustering delineates 12 discrete cellular clusters representing major cell types including epithelial, endothelial, fibroblasts, and monocytes. (c) Re-UMAP shows the distribution of epithelial cells extracted from whole cells across samples. (d) Categorization of epithelial cells into tumor-associated (red) and normal (green) phenotypes based on sample origin. (e) Enriched KEGG pathways in tumor-associated epithelial populations. (f-g) UMAP and violin plots showing the distribution of cells of HK2; (h-i) UMAP and violin plots showing the distribution of cells of ASCT2; (j) Violin plots showing the distribution of cells of CD44. Statistical significance was calculated via Student’s t-test, ****P*＜0.001.

**Figure S8.** Validation of the transcription levels of HKII, ASCT2 and CD44 in TCGA-CESC cohort. Statistical significance was calculated via Student’s t-test, **P*＜0.05.

**Figure S9.** HKⅡ expression level based on immunohistochemical assay using the cervical tissue microarray (HUteS30PG01-1). Scale bars: 500 μm. *Note: The same tissue microarray was used for both Figure S9 and S10.*

**Figure S10.** ASCT2 expression level based on immunohistochemical assay using the cervical tissue microarray (HUteS30PG01-1). Scale bars: 500 μm. *Note: The same tissue microarray was used for both Figure S9 and S10.*

**Table S1.** Detailed clinical characteristics of 15-paired cervical carcinoma /adjacent tissue microarray.

| Patient ID number | Sex | Lesin location | Histological type | Tumor grade | Age at surgery |
| --- | --- | --- | --- | --- | --- |
| J03A0136 | Female | Cervix | P/T | Grade 2 | 42 |
| J03A0204 | Female | Cervix | P/T | Unknown | Unknown |
| J03A0410  J03A1009  J03A0377  J03A0980  J03A0190  J03A0972  J03A0981  J03A0334  J03A0973  J03A1041  J03A0329  J03A1010  J03A1267 | Female  Female  Female  Female  Female  Female  Female  Female  Female  Female  Female  Female  Female | Cervix  Cervix  Cervix  Cervix  Cervix  Cervix  Cervix  Cervix  Cervix  Cervix  Cervix  Cervix  Cervix | P/T  P/T  P/T  P/T  P/T  P/T  P/T  P/T  P/T  P/T  P/T  P/T  P/T | Grade 2  Grade 2  Grade 2  Grade 3  Grade 2  Grade 1  Grade 1  Grade 3  Grade 2  Grade 2  Grade 2  Grade 2  Grade 2 | 30  49  32  43  65  52  40  54  45  44  41  41  58 |

P/T: para-tumor/tumor

**Figure S11.** Western blot analysis of HKII and ASCT2 expression in 12-paired human surgical specimens. N, normal tissues; T, tumor tissues.

**Table S2.** Detailed clinical characteristics of 12-paired human surgical specimens.

| Patient ID number | Sex | Lesin location | Histological type | Tumor grade | Age at surgery |
| --- | --- | --- | --- | --- | --- |
| Sample1 | Female | Cervix | N/T | Grade 2 | Absent |
| Sample2 | Female | Cervix | N/T | Grade 1 | 39 |
| Sample3  Sample4  Sample5  Sample6  Sample7  Sample8  Sample9  Sample10  Sample11  Sample12 | Female  Female  Female  Female  Female  Female  Female  Female  Female  Female | Cervix  Cervix  Cervix  Cervix  Cervix  Cervix  Cervix  Cervix  Cervix  Cervix | N/T  N/T  N/T  N/T  N/T  N/T  N/T  N/T  N/T  N/T | Grade3  Grade 2  Grade 3  Grade 2  Grade 2  Grade 1  Grade 3  Grade 2  Grade 3  Grade 2 | 50  51  55  62  44  38  47  59  48  64 |

N/T: normal/tumor

**Figure S12.** HKII expression level based on immunohistochemical assay using 5-paired mouse tissues. Scale bars: 1 mm. *Note: The same tissues were used for both Figure S12 and S13.*

**Figure S13.** Immunohistochemical staining and quantification of ASCT2 and CD44. (a) ASCT2 and (b) CD44 staining in paired mouse tissues (n=5). Scale bars: 1 mm. (c) The tumor-to-normal (T/N) expression ratio for each target was quantified using the H-Score from the same samples. Data were analyzed by a paired t-test, **P*＜0.05. *Note: The same tissues were used for both Figure S12 and S13.*

**Figure S14.** Western blot analysis of HKII and ASCT2 expression in 5-paired mouse surgical specimens. N, normal tissues; T, tumor tissues.

**Figure S15.** The protein levels of the indicated proteins in normal cervical epithelial cell line (End1/E6E7) compared to cervical cancer cell lines (C33A, HeLa, SiHa, ME180 and MS751).

**Figure S16.** Immunofluorescence staining of HKII, ASCT2 and CD44 in End1/E6E7 (normal cervical epithelial cell line) versus SiHa (cervical cancer cell line). Scale bars: 100 μm.

**Figure S17.** ^1^H NMR spectra of a) DSPE-PEG_2K_ and b) DSPE-PEG_2K_-Gln.

**Figure S18.** MOLDI-TOF-MS analysis of (a) DSPE-PEG_2K_ and (b) DSPE-PEG_2K_-Gln.

**Figure S19.** The cellular internalization of Cy7. Scale bars:100 μm. Data are represented as mean ±SD (n＝3). Statistical significance was calculated via one-way ANOVA, ****P*＜0.001.

**Figure S20.** Mean fluorescence intensity of intracellular Cy7 in different groups. Scale bars:100 μm. Data are represented as mean ±SD (n＝3). Statistical significance was calculated via one-way ANOVA, ****P*＜0.001.

**Figure S21.** The protein levels of HKⅡ in SiHa cells in each group.

**Figure S22.** Quantitative analysis of JC-1 fluorescence intensity in the mitochondrial membrane potential detection assay.

**Figure S23.** Quantification of intracellular ROS in SiHa cells receiving different treatments. Data are represented as mean ±SD (n＝3). Statistical significance was calculated via one-way ANOVA, ns means not significant, ****P*＜0.001.

**Figure S24.** Viability of SiHa cells following various treatments for 24 hours. Data are represented as mean ±SD (n＝3). Statistical significance was calculated via one-way ANOVA, ns means not significant, ****P*＜0.001.

**Figure S25.** Hemolysis assay with different concentrations of GL. Data are represented as means ±SD (n＝3).

**Figure S26.** Individual tumor growth kinetics (n＝5).

**Figure S27.** Expression of HKⅡ was assessed in tumor tissues in each group. Scale bars: 50 μm. Statistical significance was calculated via one-way ANOVA, ns means not significant, ****P*＜0.001.

**Figure S28.** The ATP content in tumors in each group was detected with an ATP assay kit. Statistical significance was calculated via one-way ANOVA, ns means not significant, ****P*＜0.001.

**Figure S29.** Representative immunofluorescence images and quantitative analysis of oxidative damage markers 4-HNE (a) and 8-OHdG (b) in tumor tissues from different experimental groups. Scale bars: 50 μm. Statistical significance was calculated via one-way ANOVA, ns means not significant, ****P*＜0.001.

**Figure S30.** Western analysis of Cyt-C, Bax and Bcl-2 within tumors in each group.

**Figure S31.** Quantification of mean fluorescence levels of TUNEL and KI-67 within tumor tissues. All data are represented as means ±SD (n＝5). Statistical significance was calculated via one-way ANOVA, ns means not significant, ****P*＜0.001.

**Figure S32.** Body weight changes in mice during the course of treatment. All data are represented as means ±SD (n＝5).

**Figure S33.** Representative H&E-stained sections of major organs. Scale bars: 50 μm.

**Figure S34.** Quantification of the relative expression of P-gp, Survivin and MRP2 in SiHa and SiHa-DR cells. Data are represented as mean ±SD (n＝6). Student’s *t*-test, ****P*＜0.001.

**Figure S35.** The chemoresistant phenotype did not alter the expression of HKⅡ and ASCT2 in SiHa cells. Data are represented as mean ±SD (n＝6). Student’s *t*-test, ****P*＜0.001.

**Figure S36.** Characterization of GL@DDP and GL@LND@DDP. (a, b) The hydrodynamic diameters (a) and zeta potentials (b) were measured. Data are represented as mean ±SD (n＝3).

**Figure S37.** Viabilities of SiHa-DR cells were measured after 24 hours of incubation with different GL concentrations. Data are represented as mean ±SD (n＝3). Statistical significance was calculated via one-way ANOVA, ns means not significant.

**Figure S38.** Cellular uptake of Cy7 in SiHa-DR cells in different groups. Scale bars:100 μm. Data are represented as mean ±SD (n＝3). Statistical significance was calculated via one-way ANOVA, ****P*＜0.001.

**Figure S39.** Fluorescent images and quantifications of internalized Cy7 in different groups. Scale bars:100 μm. Data are represented as mean ±SD (n＝3). Statistical significance was calculated via one-way ANOVA, ****P*＜0.001.

**Figure S40.** Quantification of the relative expression of MRP2 in SiHa-DR cells after indicated treatments. Data are represented as mean ±SD (n＝3). Statistical significance was calculated via one-way ANOVA, ****P*＜0.001.

**Figure S41.** Fluorescent images and quantifications of MRP2 in the SiHa-DR cells after indicated treatments. Data are represented as mean ±SD (n＝3). Statistical significance was calculated via one-way ANOVA, ***P*＝0.01, ****P*＜0.001.

**Figure S42.** Quantification of the relative expression of G6PD in SiHa-DR cells after indicated treatments. Data are represented as mean ±SD (n＝3). Statistical significance was calculated via one-way ANOVA, **P*＜0.05, ****P*＜0.001.

**Figure 43.** Fluorescent images and quantifications of γ-H2AX foci in the SiHa-DR cells after indicated treatments. Data are represented as mean ±SD (n＝3). Statistical significance was calculated via one-way ANOVA, ns means not significant, **P*＜0.05, ****P*＜0.001.

**Figure S44.** Fluorescent images and quantifications of JC-1 in SiHa-DR cells after indicated treatments. Scale bars:100 μm. Data are represented as mean ± SD.

**Figure S45.** Fluorescent images and quantifications of ROS in the SiHa-DR cells after indicated treatment. Scale bars:100 μm. Data are represented as mean ±SD (n＝3). Statistical significance was calculated via one-way ANOVA, ***P*＜0.01, ****P*＜0.001.

**Figure S46.** Quantitative analysis of apoptosis rate of SiHa-DR cells receiving different treatments. Data are represented as means ±SD (n＝3). Statistical significance was calculated via one-way ANOVA, ns means not significant, ****P*＜0.001.

**Figure S47.** Cell viability of SiHa-DR cells after different treatments for 24 h (a) and 48 h (b). Data are represented as mean ±SD (n＝3). Statistical significance was calculated via one-way ANOVA, ****P*＜0.001.

**Figure S48.** Representative images and diameter changes of SiHa-DR MCTSs after incubation with various formulations at different times. Scarle bars: 200 μm. Data are represented as means ±SD (n＝3). Statistical significance was calculated via one-way ANOVA, ns means not significant, **P*＜0.05, ***P*＜0.01, ****P*＜0.001.

**Figure S49.** Serum biochemical indexes in SiHa-DR-bearing mice receiving different treatments. Data are represented as means ±SD (n＝3). Statistical significance was calculated via one-way ANOVA, ns means not significant.

**Figure S50.** H&E staining images of major organs excised from SiHa-DR-bearing mice receiving diverse treatments. Scale bars: 50 μm.

1. Corresponding authors: mj2024@xjtu.edu.cn (Jia Ma); liangliangdai@nwpu.edu.cn (Liangliang Dai); Ayshamgul Hasim (axiangu@xjmu.edu.cn)
